# Intra-tumoral microbiota: Key modulators of tumor immunity and therapeutic potential

**DOI:** 10.1016/j.gendis.2025.101963

**Published:** 2025-12-05

**Authors:** Junju He, Hui Tan, Yanru Qiu, Yuchao Dan, Qian Wan, Lan Li, Jie Wu, Qibin Song, Hongbin Chen, Bin Xu

**Affiliations:** a Cancer Center, Renmin Hospital of Wuhan University, Wuhan, Hubei 430060, China; b Department of Radiology, Renmin Hospital of Wuhan University, Wuhan, Hubei 430060, China; c Department of Pulmonary and Critical Care Medicine, Renmin Hospital of Wuhan University, Wuhan, Hubei 430060, China; d Oncology Department, Jianli People’s Hospital, Jingzhou, Hubei 433300, China

**Keywords:** Cancer therapeutics, Intra-tumoral microbiota, Microbial heterogeneity, Tumor immunity, Tumor microenvironment

## Abstract

The tumor microenvironment is increasingly recognized as a complex ecosystem in which the intra-tumoral microbiota (comprising bacteria, fungi, viruses, *etc*.) plays a pivotal, underappreciated role in cancer biology. This review systematically summarizes the latest research into the origins, diversity, and functional mechanisms of intra-tumoral microbiota, emphasizing their dual roles in tumor formation, progression, and response to treatment. Using high-throughput sequencing, spatial multi-omics, and integrative bioinformatics, we identify the multifactorial origins of tumor-resident microbiota, including translocation from adjacent tissues, hematogenous dissemination, and viral genomic integration. We also examine the significant inter- and intra-tumoral microbial heterogeneity influenced by anatomical, environmental, and immunological factors, and the tissue-specific functional effects of important microbial species. We detail the mechanisms by which the intra-tumoral microbiota modulate innate and adaptive immunity through pattern recognition receptor signaling, microbial antigen presentation, and microbial metabolite production, ultimately influencing tumor microenvironment composition, immune cell dynamics, and therapeutic efficacy. Finally, we critically evaluate emerging microbiota-targeted therapeutic strategies, including engineered bacteria, antibiotics, bacteriophages, and oncolytic viruses, while outlining the technical, mechanistic, and regulatory challenges that hinder their clinical translation. Our synthesis highlights the need for rigorous multi-omics profiling, causal inference, and smart delivery systems to exploit the intra-tumoral microbiota for precision oncology. This paradigm shift offers unprecedented opportunities for personalized diagnosis and therapy, marking a new frontier in cancer research and treatment.

## Introduction

The tumor microenvironment (TME) is a highly complex and dynamic ecosystem composed of tumor cells, various non-malignant cells (such as immune cells and fibroblasts), and the extracellular matrix. For a long time, our understanding of the TME has focused primarily on host-derived components. However, with the rapid advancements in high-throughput sequencing technologies and bioinformatic analyses, the intra-tumoral microbiota, a key player that was once overlooked, has come to the forefront of research.^1^^,^^2^ This has challenged the long-held belief that tumor interiors are sterile environments, revealing that microbial communities, including bacteria, fungi, and viruses, exist within tumor tissues and act as integral parts of this ecosystem.^2^^,^^3^ These communities engage in dynamic and profound interactions with tumor cells, immune cells, and other TME components, collectively forming a complex regulatory ecological network.

Emerging evidence demonstrates that intra-tumoral microbiota play a pivotal, “double-edged sword” role in tumorigenesis, progression, metastasis, and even the response to therapy.^4^^,^^5^ Some microbes, such as *Helicobacter pylori* (*H. pylori*) and *Fusobacterium nucleatum* (*F. nucleatum*), can directly or indirectly promote tumor cell proliferation, invasion, and immune evasion by secreting toxins, producing metabolic byproducts, or inducing chronic inflammation, thus acting as “accomplices” in carcinogenesis.^6^^,^^7^ Conversely, other microbes, such as *Bifidobacterium*, can act as “guardians” by activating anti-tumor immune responses and enhancing the efficacy of treatments like immune checkpoint inhibitors (ICIs).^8^^,^^9^ This complex duality has rapidly positioned the intra-tumoral microbiota as a research hotspot at the intersection of oncology and immunology, offering an unprecedented opportunity to understand cancer biology from a novel micro-ecological perspective.

However, despite this rapid progress, our understanding of the intra-tumoral microbiota remains in its infancy. The precise origins of these microbiota (whether from adjacent tissues, the circulatory system, or other sources), the core drivers of their high heterogeneity across different tumor types and even within different regions of the same tumor, and the exact molecular mechanisms governing their interactions with the host immune system are still not fully understood.^2^^,^^10^ Furthermore, challenges such as the low biomass of intra-tumoral microbiota, the susceptibility of samples to contamination, and the predominantly correlational (rather than causal) nature of existing studies pose significant threats to the reliability and universality of research findings.^1^^,^^11^ These knowledge and technical gaps are the core scientific challenges in the field, severely constraining our ability to translate these fundamental discoveries into reliable clinical diagnostic markers and novel precision therapeutic strategies.

To address these challenges and advance the field, this review aims to systematically summarize and combine the latest research on intra-tumoral microbiota.^12^^,^^13^ We will take a logical approach, moving from the macroscopic to the mechanistic. First, we will explore the potential origins and complex heterogeneity of intra-tumoral microbiota.^2^^,^^10^ Second, we will focus on the multi-dimensional networks and intricate mechanisms through which they regulate innate and adaptive tumor immunity, which is central to understanding their function.^4^^,^^14^ Third, we will critically evaluate the potential and challenges of various emerging therapeutic strategies based on intra-tumoral microbiota, including engineered bacteria, antibiotics, bacteriophages, and oncolytic viruses.^15^^,^^16^ Ultimately, this review will offer perspectives on future research directions, providing a comprehensive roadmap for in-depth exploration and clinical translation to establish a new paradigm in personalized, microbiota-based oncology.

## Current research landscape of intra-tumoral microbiota

Recent studies have identified a wide array of microbial communities within tumors, varying by cancer type and anatomical location. Predominant bacterial phyla include *Proteobacteria*, *Firmicutes*, and *Actinobacteria*, with specific genera such as *F. nucleatum* in colorectal cancer (CRC), *H. pylori* in gastric cancer, and *Pseudomonas* in pancreatic cancer showing significant associations with tumor progression. Viruses like *Epstein–Barr virus* (*EBV*), *human papillomavirus* (*HPV*), and *Hepatitis B Virus* (*HBV*) are well-documented in nasopharyngeal carcinoma, cervical carcinoma, and hepatocellular carcinoma, respectively. These microbiota influence critical biological processes, including inflammation, immune suppression, and metabolic reprogramming. Table 1 summarizes the major intra-tumoral microbiota, associated cancers, and their reported roles in tumor biology, providing a foundation for understanding their therapeutic potential.

**Table 1:** Major intra-tumoral microbiota, associated cancers, and their roles in tumor biology.

Cancer type	Dominant microbiota	Biological processes	Key references
Nasopharyngeal carcinoma	*Fusobacterium nucleatum*	Tumor progression, metastasis, and radioresistance	Guo et al, 2024^17^
	*Proteobacteria*	Immune evasion and inflammation	Zhang et al, 2024^18^
	*Corynebacterium*, *Staphylococcus*	T-lymphocyte infiltration	Qiao et al, 2024^19^
	*EBV*	Tumorigenesis and immune evasion	Yuan et al, 2025^20^ Caetano et al, 2020^21^ Chen et al, 2019^22^
Colorectal cancer	*Campylobacter jejuni*	Promote intestinal inflammation	He et al, 2019^23^
	*Escherichia coli*	Autophagy and inflammasome activation	Lucas et al, 2020^24^ Salesse et al, 2024^25^
	*Enterotoxigenic Bacteroides fragilis*	Cancer stemness	Liu et al, 2020^26^
	Tumor-resident *Escherichia coli E*	Premetastatic niche	Bertocchi et al, 2021^27^
	*Fusobacterium nucleatum*	Chemoresistance	LaCourse et al, 2022^28^
	*Fusobacterium nucleatum*	Enhance tumor metastasis and myeloid cell recruitment	Galeano Niño et al, 2022^29^
	Colibactin-producing *E. coli (CoPEC)*	Epithelial–mesenchymal transition, tumor cell stemness, and chemoresistance	Dalmasso et al, 2024^30^
		Chemoresistance	de Oliveira Alves et al, 2024^31^
		Intestinal inflammation, tumorigenesis	Thakur et al, 2025^32^
	*Peptostreptococcus anaerobius*	Immunosuppression	Liu et al, 2024^33^
	*Escherichia coli*	Immune escape and tumor metastasis	Gu et al, 2024^34^
	*Fusobacterium nucleatum animalis C2*	Tumorigenesis	Zepeda-Rivera et al, 2024^35^
	*Fusobacterium nucleatum*	Inhibit NK cell activity	Chamutal et al, 2015^36^
	*Fusobacterium nucleatum*	Inhibit T cell function	Johanna et al, 2021^37^
Non-small cell lung cancer	*Aspergillus sydowii*	Immunosuppression	Liu et al, 2023^38^
	*Akkermansia muciniphila*	Metabolic reprogramming	Zhu et al, 2023^39^
	*Acidovorax temperans*	Tumorigenesis and inflammation	Stone et al, 2024^40^
	*Roseburia*	Tumor metastasis	Ma et al, 2024^41^
	*Escherichia coli*	Enhance immunotherapy response	Elkrief et al, 2024^42^
Liver cancer	*Paraburkholderia fungorum*	Metabolic reprogramming	Chai et al, 2023^43^
	*Brevibacillus parabrevis*	Inhibit NK cell ferroptosis	Pan et al, 2025^44^
	*Cutibacterium*	Immunosuppressive, chronic inflammatory microenvironment	Liu et al, 2024^45^
	*Malassezia*	Immune escape and tumor proliferation	Shen et al, 2025^46^
	*Enterococcus gallinarum*	Inflammation	Yang et al, 2022^47^
	*HBV*	Chronic inflammation, viral DNA integration, oncogenic viral proteins expression, epigenetic regulation abnormalities, and immune microenvironmental modulation	Levrero et al, 2016^48^ Iannacone et al, 2021^49^ Zoulim et al, 2021^50^
	*HCV*	Chronic inflammatory response, oncogenic effects of viral proteins, genetic/epigenetic abnormalities, and immune microenvironmental modulation	Martinello et al, 2023^51^ Torres et al, 2017^52^ Hwang et al, 2024^53^
Cervical cancer	*Pseudomonas*	Tumor proliferation and metastasis	Guo et al, 2025^54^
	*Lactobacillus iners*	Chemoradiation resistance	Colbert et al, 2023^55^
	*HPV*	Chronic inflammation, viral DNA integration, oncogenic viral proteins, epigenetic abnormalities, and immune microenvironmental modulation	Malagón et al, 2024^56^ Cohen et al, 2019^57^ Yuan et al, 2021^58^
Melanoma	*Fusobacterium nucleatum* *Fusobacterium* *Staphylococcus* *Actinomyces* *Acinetobacter*	Bacterial peptides presented by MHC-I/II activate CD8^+^/CD4^+^ T cells	Kalaora et al, 2021^59^
	*Neospora caninum*	Induce tumor cell death and activate Th1-type immune response	Li et al, 2022^60^
	*Lactobacillus reuteri*	Promote interferon-γ-producing CD8 T cells and bolster immune checkpoint inhibitors	Bender et al, 2023^61^
Ovarian cancer	*Propionibacterium acnes*	Chronic inflammation	Huang et al, 2024^62^
	*Bacillus velezensis*	Induce apoptosis and inhibit angiogenesis	Sreejesh et al, 2023^63^
Head and neck tumor	*Fusobacterium nucleatum*	Autophagy, epithelial–mesenchymal transition, and metastasis	Chen et al, 2024^64^
	*Streptococcus mutans*	Tumor progression and immunotherapy resistance	Zhou et al, 2024^65^
	*Lactobacillus johnsonii*	Tumor progression and metastasis	Xie et al, 2025^66^
	*HPV*	Drive tumorigenesis and progression through viral gene integration, oncogenic viral proteins expression, epigenetic abnormalities, and immune microenvironmental modulation	Malagón et al, 2024^56^ Taberna et al, 2017^67^ Zandberg et al, 2013^68^
Esophageal squamous cell carcinoma	*Fusobacterium nucleatum*	Chemoresistance	Kensuke et al, 2019^69^
	*Streptococcus*	Enhance chemoimmunotherapy response by promoting CD8^+^ cell infiltration, inhibiting Tregs, and enhancing immune activation	Wu H et al, 2023^70^
	*Fusobacterium nucleatum*	Immunotherapy resistance and immune escape	Li Y et al, 2023^71^
	*Fusobacterium periodonticum*	Epithelial–mesenchymal transition and lipid metabolic reprogramming	Sun et al, 2024^72^
	*Streptococcus mutans*	Tumor progression and immunotherapy resistance	Zhou et al, 2024^65^
Prostate cancer	*Escherichia coli CP1*	Enhance the efficacy of immune checkpoint inhibitors	Anker et al, 2018^73^
	*Escherichia coli CP1*	Induce long-term chronic inflammation in the prostate and accelerate cancer progression and infiltrative transformation	Brian et al, 2018^74^
	*Cutibacterium acnes*, *Escherichia coli*	Chronic inflammation	Holger et al, 2024^75^
	*Cutibacterium acnes*	Inflammation and tumorigenesis	Fu et al, 2025^76^
	*Escherichia coli*	Induce oncogene fusions	Eva et al, 2021^77^
Breast cancer	*Staphylococcus*, *Lactobacillus*	Tumor metastasis	Fu et al, 2021^78^
	*Pseudomonas*	Promote tumor progression and chemosensitivity	Akiko et al, 2020^79^
	*Fusobacterium nucleatum*	Small extracellular vesicles facilitate tumor growth and metastasis	Li et al, 2023^80^
	*Fusobacterium nucleatum*	Inhibit NK cell-mediated cancer cell killing	Johanna et al, 2023^81^
	*Fusobacterium nucleatum*	Promote tumor growth and metastasis	Zhao et al, 2023^82^
	*Fusobacterium nucleatum*	Promote tumor growth and lung metastasis	Lishay et al, 2020^83^
	Enterotoxigenic *Bacteroides fragilis*	Promote cancer stemness and chemoresistance	Ma et al, 2024^84^
	Enterotoxigenic *Bacteroides fragilis*	Promote tumorigenesis and metastatic progression	Sheetal et al, 2021^85^
	*Faecalibacterium prausnitzii*	Suppress tumor proliferation and invasion	Ma et al, 2020^86^
	*Escherichia coli*	Metabolic reprogramming	Reem et al, 2023^87^
	*Escherichia coli*	Induce epithelial–mesenchymal transition and stemness in normal breast epithelial cells	Jamilah et al, 2024^88^
	*Staphylococcus aureus*	Induce anti-tumor immunity and suppress cancer aggressiveness	Giancarla et al, 2022^89^
	*Staphylococcus aureus*	Inhibit cancer cell adhesion and bone metastasis	Darius et al, 2007^90^
	*Staphylococcus aureus*	Tumor metastasis	Zhao et al, 2007^91^
	*Stenotrophomonas maltophilia*	Promote CD8^+^ T cell migration and activation	Zhang et al, 2025^92^
Gastric cancer	*Helicobacter pylori*	Promote carcinogenesis and tumor progression through multiple mechanisms, including chronic inflammatory, virulence factor effects, genetic/epigenetic abnormalities and immune microenvironmental modulation	Usui et al, 2023^93^ Wizenty et al, 2025^94^ Duan et al, 2025^95^
	*EBV*	Promote carcinogenesis and tumor progression by activation of tumorigenic pathways, immune escape, genetic and epigenetic abnormalities, and immune microenvironmental modulation	Naseem et al, 2019^96^ Chen et al, 2021^97^ Zhou et al, 2025^98^ Wen et al, 2024^99^
	*Methylobacterium*	Decrease TGFβ expression and CD8^+^ tissue-resident memory T cells	Peng et al, 2022^100^
	*Streptococcus anginosus*	Promote tumor cell proliferation, migration, and immune escape	Yuan et al, 2024^101^
	*Fusobacterium nucleatum*	Recruit tumor-associated neutrophils to promote tumor progression and immune evasion	Zhang et al, 2025^102^
	*Streptococcus*	Inhibit M2-type macrophage polarization and infiltration	Yuan et al, 2025^103^
Pancreas cancer	*Proteobacteria*, *Actinobacteria*	Drive macrophage M2 polarization, cause immune tolerance, and inhibit T-cell activation	Pushalkar et al, 2018^104^
	*Gammaproteobacteria*	Gemcitabine chemotherapy resistance	Geller et al, 2017^105^
	*Malassezia*	Activating the complement C3 pathway promotes carcinogenesis and tumor progression	Aykut et al, 2019^106^
	*Malassezia*, *Alternaria*	Drive Th2 and ILC2 cell recruitment and activation to form a type II immunosuppressive microenvironment	Alam et al, 2022^107^
	*Porphyromonas gingivalis*	Promote carcinogenesis and progression, and establish an immunosuppressive and pro-inflammatory microenvironment	Tan et al, 2022^108^
	*Fusobacterium nucleatum*	Promote cancer cell proliferation and migration	Udayasuryan et al, 2022^109^
	*Pseudomonas fluorescens*	Inhibit cancer cell proliferation and enhance apoptosis	Gao et al, 2024^110^
	*Bacillus coagulans*	Inhibit tumor progression	Zhang et al, 2024^111^
	*Micrococcus*	Chronic inflammation	Lu et al, 2021^112^
	*Peptostreptococcus*	Promote an immune-tolerant microenvironment and tumor progression	Wang et al, 2024^113^

## Origins of intra-tumoral microbiota

The origin of intra-tumoral microbiota is a complex and multifactorial process, rather than stemming from a single, universal source.^3^ It is increasingly understood as a dynamic interplay between microbial seeding from various body sites and subsequent selection and adaptation within the unique ecological niche of TME.^2^^,^^3^ Current evidence supports several interconnected pathways of colonization, which are not mutually exclusive but often act in concert.^2^ These pathways include translocation from anatomically proximal sites, dissemination through the bloodstream, and the deep integration of oncogenic viruses that drive tumor formation.^3^^,^^114^

### Translocation from anatomically proximal sites via compromised barriers

The most direct route for microbiota to enter tumors is through translocation from adjacent, microbiota-rich tissues, especially when epithelial or mucosal barriers are damaged. This pathway is most evident in cancers that arise in or near mucosal surfaces, such as the gastrointestinal tract, the oral cavity, and the respiratory system.^3^^,^^14^

**Figure 1:**
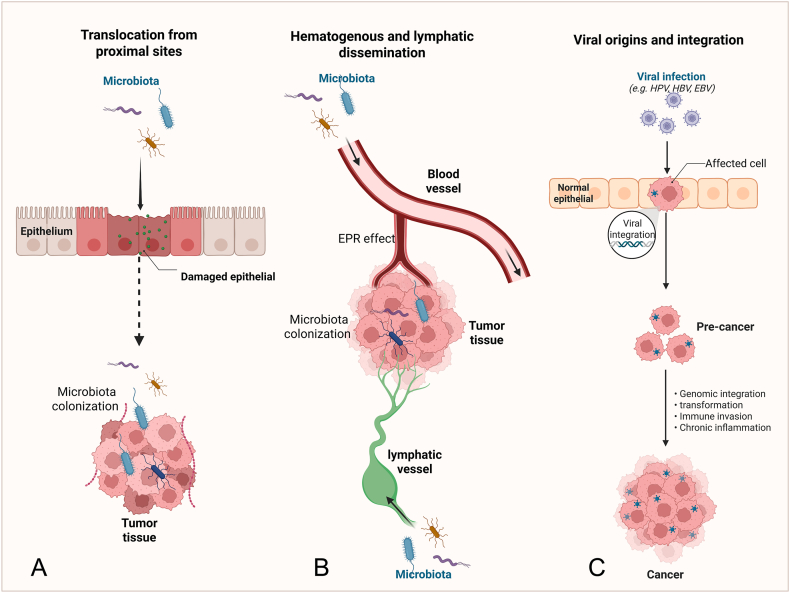
The schematic diagram of origins of intra-tumoral microbiota. **(A)** Microbiota can invade tumor tissues directly from neighboring, microbe-rich sites when epithelial or mucosal barriers are disrupted. **(B)** Microbiota can reach distant, normally sterile tumors via blood or lymphatic spread. **(C)** Oncogenic viruses integrate their genomes into host cells, driving tumorigenesis through oncogene expression, immune evasion, and persistent infection. EPR, enhanced permeability and retention; HPV, human papillomavirus; HBV, hepatitis B virus; EBV, Epstein–Barr Virus.

Tumor progression itself often leads to the physical disruption of these barriers, creating portals for microbial infiltration.^5^ For example, in CRC, the intestinal epithelial barrier is frequently impaired, enabling gut commensals to invade the tumor tissue directly.^7^ This explains why the microbial composition of CRC tissues often reflects, yet differs from, the surrounding luminal gut microbiota.^7^ Key species such as *F. nucleatum*, *Bacteroides fragilis*, and certain *Escherichia coli* (*E. coli*) strains are significantly enriched in CRC tissues compared with adjacent normal mucosa.^7^^,^^115^^,^^116^ These bacteria are not passive bystanders; they actively contribute to a pro-tumorigenic environment by inducing chronic inflammation, producing genotoxins (*e.g.*, colibactin from *E. coli*), and modulating local immune responses.^115^^,^^116^ Similarly, in oral squamous cell carcinoma, microbiota from the oral cavity, such as *Porphyromonas gingivalis*, can infiltrate tumor tissues, where they promote tumor progression through chronic inflammation and immune evasion mechanisms.^117^ In this context, the “adjacent tissue” hypothesis and the “compromised barrier” hypothesis are two facets of the same core mechanism: local invasion driven by proximity and opportunity (Fig. 1A).

### Hematogenous and lymphatic dissemination to distant tumors

Microbiota can also reach tumors in sterile anatomical locations, such as the breast, pancreas, or bone, via the circulatory and lymphatic systems.^2^^,^^118^ This “hematogenous dissemination” pathway posits that microbiota can transiently enter the bloodstream (bacteremia) or lymphatic fluid from sites like the gut or oral cavity, particularly when local barriers are disrupted.^1^^,^^2^

Once in circulation, these microbiota can colonize distant tumors, which act as selective filters.^2^ The tumor vasculature is often abnormal and leaky, a phenomenon known as the enhanced permeability and retention effect, which can facilitate the extravasation of microbiota into the tumor stroma.^119^ More importantly, specific molecular interactions can mediate tumor tropism. A landmark example is the colonization of colorectal cancer and breast cancer by *F. nucleatum*.^83^^,^^120^ Its surface adhesin, fibroblast activation protein 2 (Fap2), specifically binds to the Gal-GalNAc sugar moiety, which is overexpressed on the surface of many cancer cells.^120^ This binding anchors the bacterium to the tumor, enabling stable colonization and subsequent pro-tumorigenic activities.

Furthermore, the TME provides a fertile ground for microbial survival and proliferation.^2^^,^^3^ The characteristic hypoxia, nutrient richness from necrotic regions, and profound immunosuppression within many tumors create a unique niche that can support the growth of specific anaerobic or facultative anaerobic bacteria, which might otherwise be cleared by the immune system in healthy tissues.^3^^,^^12^ Thus, the tumor is not merely a passive recipient but an active selector of its microbial inhabitants (Fig. 1B).

### Special focus on viral origins: HPV as a model of complex integration

While the above hypotheses broadly apply to bacteria and fungi, viral origins warrant distinct consideration due to their obligate intracellular nature and genomic integration capabilities.^121^ Viruses often originate from adjacent tissues or systemic spread but establish deeper tumor interactions through mechanisms such as latency, oncogene expression, and host genome alteration.^121^^,^^122^ *HPV* in cervical cancer is a paradigmatic model that illustrates this complexity.

*HPV*, primarily types 16 and 18, infiltrates via compromised cervical mucosa during sexual transmission, integrating its DNA into host epithelial cells.^123^ The core mechanisms include: i) Genomic integration, where viral E6 and E7 oncogenes disrupt tumor suppressors; E6 ubiquitinates p53, inhibiting apoptosis; E7 binds to Rb, deregulating cell cycle^124^^,^^125^; ii) Viral protein expression in tumor cells, sustaining transformation and immune evasion (*e.g.*, E5 down-regulates MHC-I)^126^; iii) Chronic inflammation and co-factors (*e.g.*, smoking) enhancing persistence.^127^ Longitudinal studies show integration correlates with progression from dysplasia to carcinoma, with viral load predicting outcomes.^128^ Compared with bacterial origins, HPV integration is more “intrinsic”, directly driving oncogenesis rather than opportunistic colonization.^122^ Similar patterns occur in *EBV* (lymphomas) and *HBV*, where episomal or integrated forms modulate TME immunity.^129^^,^^130^ This model highlights the unique nature of viruses and informs therapies like *HPV* vaccines^131^ (Fig. 1C).

In summary, the origins of the intra-tumoral microbiota are best understood as a multi-step, ecological process. It begins with microbial seeding, which can occur through local translocation from adjacent sites or systemic dissemination from distant reservoirs. This is followed by selective colonization, where the unique biological features of the TME—including its specific cell-surface receptors (*e.g.*, Gal-GalNAc), hypoxia, nutrient profile, and immunosuppressive state—determine which microbiota can survive and thrive. In the case of oncoviruses, the microbiota is the inciting agent, reprogramming the host cell to create its own malignant niche. Therefore, a comprehensive understanding requires appreciating that these pathways are interconnected and that the final composition of a tumor’s microbiome is the result of a dynamic dialogue between the invading microorganism and the evolving tumor ecosystem. Future research using advanced spatial and multi-omics technologies will be crucial to further dissect these complex origin stories and their therapeutic implications.

## Intra-tumoral microbial heterogeneity

The composition, abundance, and functional roles of microbial communities within tumors exhibit remarkable heterogeneity, both across different tumor types and within individual tumors.^1^^,^^2^ This heterogeneity underscores the complexity of TME and arises from multifaceted influences, including tumor anatomical location, tissue type, host genetic and immune factors, and environmental exposures such as diet, antibiotic use, and lifestyle.^3^^,^^132^^,^^133^ Recent advancements in metagenomic sequencing, spatial transcriptomics, and multi-omics integration have enabled a more systematic characterization of this heterogeneity, revealing tumor type-specific microbial signatures that influence immune responses, tumor progression, and therapeutic outcomes.^3^^,^^5^^,^^13^ Below, we systematically outline the microbial structures in major tumor types, their specificities, and the mechanisms underlying tissue-dependent heterogeneity.

### Systematic overview of microbial structures across major tumor types

Comprehensive studies, such as those by Nejman et al. (analyzing 1526 samples across seven cancer types via 16S rRNA sequencing) and Poore et al. (integrating TCGA data with microbiome profiling), have demonstrated distinct microbial compositions tailored to tumor histology and organ site.^1^^,^^2^ These profiles often mirror adjacent normal tissues but show enrichments or depletions linked to tumorigenesis. Here, we summarize the main findings for prominent cancers:

Colorectal cancer: CRC tumors are enriched in *F. nucleatum*, *Bacteroides fragilis*, and *E. coli*, with *Fusobacterium* comprising up to 10%–20% of the microbial load in tumors versus <1% in normal mucosa.^7^^,^^134^^,^^135^ Phylum-level dominance includes *Firmicutes* and *Bacteroidetes*, with specificities like toxin-producing strains (*e.g.*, *pks*^+^ *E. coli*) promoting DNA damage and inflammation.^116^ This composition reflects the gut’s exposure to fecal microbiota, distinguishing CRC from non-gastrointestinal cancers.

Breast cancer: Microbial profiles feature elevated *Bacillus*, *Enterobacteriaceae*, *Staphylococcus*, and *Proteobacteria*, with genera like *Lactobacillus* and *Streptococcus* linked to metastasis in murine models.^78^^,^^85^^,^^136^ Compared with healthy breast tissue, tumors show reduced microbial diversity but increased abundance of DNA-damaging species (*e.g.*, *Staphylococcus epidermidis*).^137^ Specificity arises from non-mucosal origins, often via systemic circulation, contrasting with mucosal-exposed tumors.

Pancreatic cancer: *Proteobacteria* dominate (up to 45% of bacteria), with *Pseudomonas*, *Elizabethkingia*, and *Gammaproteobacteria* enriched in tumors.^12^^,^^104^ These microbiota activate Toll-like receptor (TLR) pathways, fostering immunosuppression.^104^ Pancreatic ductal adenocarcinoma (PDAC) exhibits unique fungal components (*e.g.*, *Malassezia*) not prominent in other cancers, highlighting organ-specific mycobiome roles.^106^^,^^107^

Lung cancer: *Actinobacteria* and *Firmicutes prevail*, with *Veillonella*, *Prevotella*, and *Streptococcus* associated with tumor proliferation and ICI responses.^138^, ^139^, ^140^ Specificity includes acid-tolerant species adapted to the lung’s low-pH niches, differing from gastrointestinal microbiomes, and correlations with smoking-induced dysbiosis.^141^

Gastric cancer: Beyond *H. pylori* (an important driver via inflammation), tumors harbor *Streptococcus*, *Lactobacillus*, and *Veillonella*, modulating metabolic profiles and immune evasion.^142^, ^143^, ^144^ This profile is highly specific to the stomach’s acidic environment, with lower diversity than CRC but stronger viral components (*e.g.*, *EBV* co-infections).

Other types: Melanoma shows high *Pseudomonadota* and viral epitope diversity.^59^^,^^145^ Ovarian cancers feature *Chlamydia* and *Mycoplasma*, linked to pelvic inflammation.^146^ Brain tumors have low-biomass microbiomes with *Actinobacteria* dominance, possibly from blood–brain barrier breaches.^2^^,^^147^ These structures are not uniform; inter-patient variability is high, influenced by host factors like age and genetics.^3^

### Specificities of intra-tumoral microbiota

The specificities of these microbial communities are evident in their functional impacts. For instance, *Fusobacterium* in CRC selectively binds to tumor-expressed Gal-GalNAc via Fap2, promoting immune evasion—a mechanism highly characteristic of the colonic niche.^120^ In breast cancer, *Staphylococcus* induces DNA double-strand breaks, enhancing genomic instability.^136^ Pancreatic *Proteobacteria* confer gemcitabine resistance through enzymatic degradation, a specificity tied to the pancreas’s metabolic milieu.^105^ Lung microbiomes modulate ICI efficacy via TLR signaling, with *Veillonella* correlating with better programmed death-1 (PD-1) responses.^140^ Gastric *H. pylori* drives chronic inflammation via cytotoxin-associated gene A (CagA), uniquely up-regulating programmed cell death 1 ligand 1 (PD-L1).^148^ These traits highlight how microbial specificities align with tumor biology, often exacerbating hallmarks like immune suppression or metastasis.

### Mechanisms underlying tissue-specific heterogeneity

Tissue specificity arises from an interplay of anatomical, environmental, and TME-dependent factors.^5^^,^^149^^,^^150^

i) Anatomical and exposure factors: Mucosal tumors (*e.g.*, CRC, gastric, lung) acquire microbiota from adjacent mucosal sites or external environments, leading to high-diversity profiles enriched in oral/gut commensals.^135^^,^^144^ Non-mucosal tumors (*e.g.*, breast, brain) rely on hematogenous seeding, resulting in lower biomass and systemic-origin species.^83^^,^^147^ For example, tumor neovascularization facilitates microbial hitchhiking via circulation, as seen with *Fusobacterium* in distant metastases.^1^
ii) TME conditions: Hypoxia, nutrient gradients, and pH variations select for adapted microbiota.^3^^,^^151^ *Anaerobic Fusobacterium* thrives in CRC’s hypoxic cores, while aerobic species dominate vascular peripheries.^7^ Nutrient-rich necrotic zones support specific colonizers, and immune gradients (*e.g.*, higher tumor-infiltrating lymphocytes (TILs) at margins) exert selective pressure.^12^
iii) Host and environmental influences: Genetic factors (*e.g.*, *APC* mutations in CRC) and immune status shape microbial niches; immunosuppressive TME favors pathobionts.^3^^,^^104^ Diet and antibiotics alter compositions, with vancomycin depleting butyrate-producers and enhancing anti-tumor immunity.^152^ Mechanistically, ligand–receptor interactions (*e.g.*, bacterial adhesins binding tumor glycans) and metabolic dependencies (*e.g.*, short-chain fatty acid production in gut tumors) drive specificity.^5^^,^^153^

Intra-tumor heterogeneity further complicates this: cores show lower diversity due to hypoxia, while peripheries harbor diverse aerobes.^1^^,^^2^^,^^154^ Bullman et al. demonstrated *Fusobacterium* enrichment in CRC cores, linked to anaerobic adaptation.^7^ Despite these insights, intra-tumoral microbial heterogeneity remains understudied. Integrating spatial multi-omics (*e.g.*, metagenomics with transcriptomics) could elucidate functional implications, paving the way for microbiota-based biomarkers in precision oncology.^3^^,^^13^^,^^14^

## The intra-tumoral microbiota regulated anti-tumor immunity

The intra-tumoral microbiota is not a mere bystander within the TME but rather an active architect of its immunological landscape. Through complex signaling networks and bidirectional crosstalk, the intra-tumoral microbiota profoundly influences the activation, differentiation, and effector functions of both the innate and adaptive immune systems. Current research has elucidated several primary mechanisms of microbial-mediated immune regulation: recognition of microbial molecular patterns, presentation of microbial antigens, modulation of the immune microenvironment by metabolites, and direct regulation of immune effector cells by bacterial surface proteins and secreted vesicles.

### Innate immune regulation

**Figure 2:**
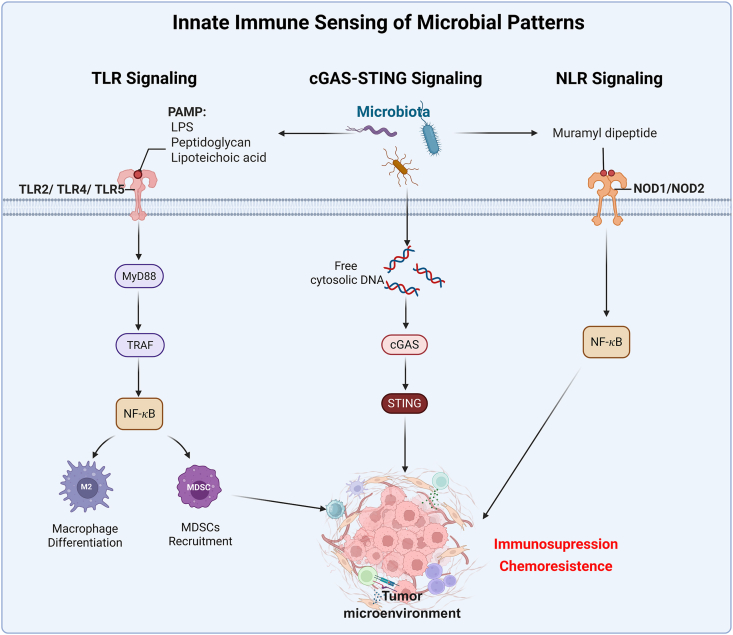
The schematic diagram of innate immune sensing of microbial patterns. TLR, Toll-like receptor; PAMP, pathogen-associated molecular patterns; LPS, lipopolysaccharides; NOD, nucleotide-binding oligomerization domain; NLR, NOD-like receptor; MDSCs, myeloid-derived suppressor cells; cGAS-STING, cyclic GMP-AMP synthase-stimulator of interferon genes; NF-κB, nuclear factor kappa-light-chain-enhancer of activated B cells.

The host’s innate immune system is the primary sensor of microorganisms within the TME. It employs various pattern recognition receptors (PRRs) to identify pathogen-associated molecular patterns (PAMPs), triggering signaling cascades that determine the nature and direction of the immune response. These core mechanisms are systematically summarized in Figure 2.

#### TLR signaling pathway

Members of the TLR family recognize a broad spectrum of microbial components. For instance, lipopolysaccharide (LPS) from Gram-negative bacteria activates TLR4, while peptidoglycan and lipoteichoic acid from Gram-positive bacteria stimulate TLR2.^155^ In pancreatic cancer, dominant intra-tumoral bacteria (*e.g.*, *B. pseudolongum)* can activate TLR2/TLR4/TLR5 signaling in macrophages, and the downstream myeloid differentiation primary response gene 88 (MyD88)-Tumor necrosis factor (TNF) receptor-associated factor 6 (TRAF6)-nuclear factor kappa B (NF-κB) signaling pathway is subsequently activated, driving macrophage differentiation towards an M2-like immunosuppressive phenotype and promoting the expansion of myeloid-derived suppressor cells (MDSCs). This ultimately cultivates an immunosuppressive TME conducive to tumor progression and chemoresistance.^104^ In CRC, *F. nucleatum* utilizes the TLR5 pathway to recruit myeloid cells, fostering a microenvironment that is both pro-inflammatory and immunosuppressive.^156^

#### cGAS-STING signaling pathway

The cGAS–STING pathway is a crucial sensor of cytosolic DNA from microbiota and plays an important role in microbe-driven anti-tumor immunity.^157^ For instance, *Bifidobacterium* can accumulate in tumors, activate STING signaling in dendritic cells (DCs), boost type I interferon (IFN) production, and enhance the anti-tumor effects of anti-CD47 therapy.^158^ However, STING activation is not always beneficial. In esophageal cancer, *F. nucleatum* can trigger STING signaling in tumor cells, leading to the secretion of chemokines that recruit immunosuppressive cells, promote tumor progression, and worsen prognosis.^159^ Thus, the impact of STING signaling depends on tumor type and microbial context. Engineered bacteria also offer new ways to activate this pathway. For example, recombinant *Salmonella* expressing C–C motif chemokine ligand 2 (CCL2) and C-X-C motif chemokine ligand 9 (CXCL9) robustly stimulated intra-tumoral cGAS–STING signaling in a mouse model, inducing immunogenic cell death, DC maturation, and T cell infiltration, and ultimately suppressing tumor metastasis.^160^

#### NOD-like receptor (NLR) signaling pathway

Degradation products of bacterial peptidoglycan, such as muramyl dipeptide, can be recognized by intracellular NLRs (*e.g.*, NOD1 and NOD2). In CRC models, NOD1 signaling has been shown to promote the expansion of MDSCs and regulate the alternative reprogramming of macrophages, collectively constructing an immunosuppressive TME.^161^

### Adaptive immune regulation

The intra-tumoral microbiota significantly impacts adaptive immunity, determining the efficacy of T cell-mediated tumor clearance. Its regulatory mechanisms include the presentation of microbial antigens and the immunomodulatory effects of secreted factors and metabolites.

### Microbial peptides: neoantigens and molecular mimicry

**Figure 3:**
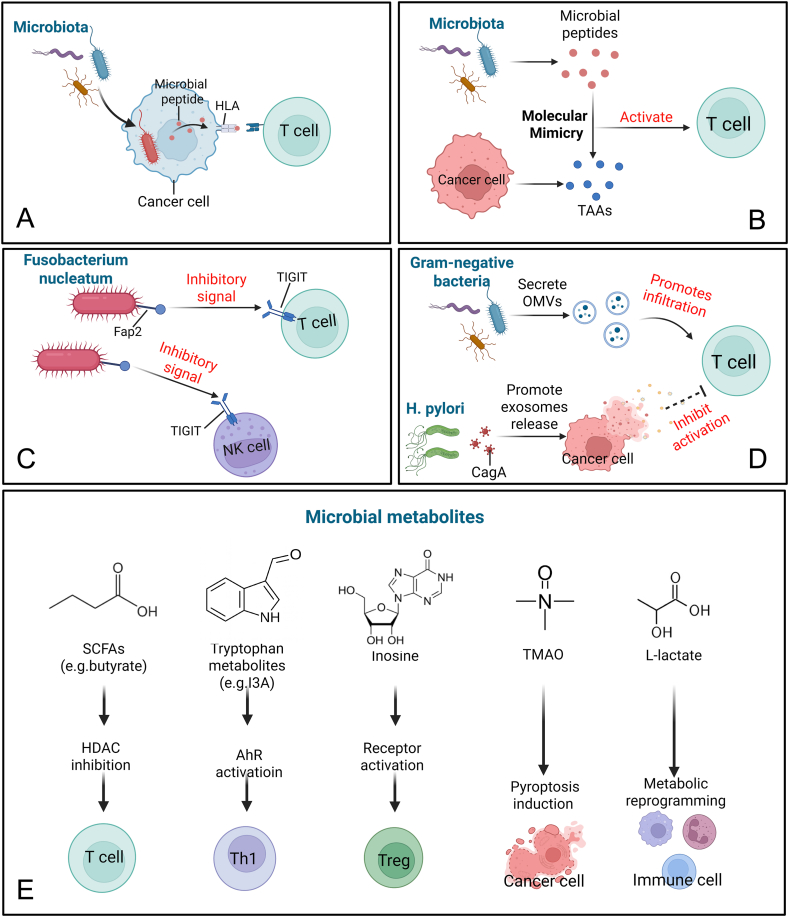
The schematic diagram of intra-tumoral microbial regulation of adaptive immunity. **(A)** Intra-tumoral microbiota generate peptides that are presented by HLA molecules, activating tumor-infiltrating lymphocytes. **(B)** Microbial peptides can mimic tumor antigens, leading to T cell cross-reactivity and anti-tumor responses. **(C)***Fusobacterium nucleatum* directly binds to the inhibitory receptor TIGIT on T and natural killer (NK) cells via its Fap2 protein, suppressing anti-tumor immune responses. **(D)** Bacterial outer membrane vesicles and virulence factors modulate the tumor immune microenvironment. **(E)** Microbial metabolites, such as short-chain fatty acids (SCFAs), tryptophan derivatives, and other small molecules, reprogram immune and tumor cell functions. EPR, enhanced permeability and retention; HPV, human papillomavirus; HBV, hepatitis B virus; EBV, Epstein–Barr Virus.
ii) Molecular mimicry and T cell cross-reactivity: Some microbial peptides share sequence or structural homology with tumor-associated antigens. This molecular mimicry can trigger a cross-reactive T cell response, wherein T cells initially primed against a microbial antigen can also recognize and attack tumor cells expressing a similar tumor-associated antigen. For example, a peptide from *Mycoplasma penetrans* can induce T cells that cross-react with the MAGE-A6 antigen,^162^^,^^163^ suggesting that microbial peptides could serve as novel targets for cancer vaccine design (Fig. 3B).

Microbial peptides are small proteins or polypeptides produced by microbiota such as bacteria, fungi, and viruses via ribosomal or non-ribosomal pathways. These peptides can act as potent antigens, stimulating T cell responses through two primary mechanisms:

i) Direct presentation via HLA: Intra-tumoral bacteria provide a rich source of exogenous peptides that can be processed and presented on human leukocyte antigen (HLA) molecules by tumor cells or antigen-presenting cells. Using mass spectrometry, Kalaora et al. identified hundreds of bacterial-derived HLA-I and HLA–II–binding peptides in melanoma, demonstrating their capacity to activate TILs and serve as a source of immune-relevant neoantigens^59^ (Fig. 3A).

#### Direct binding and secreted factors regulating immune cells

ii) Direct receptor binding: *F. nucleatum* has been shown to utilize its surface adhesion protein, Fap2, to directly bind to the inhibitory receptor T-cell immunoreceptor with Ig and ITIM domains (TIGIT), which is expressed on TILs and natural killer cells. This interaction delivers a potent inhibitory signal, effectively shielding tumor cells from immune-mediated killing^36^ (Fig. 3C).
ii) Modulation via secreted vesicles and virulence factors: Gram-negative bacteria constantly shed outer membrane vesicles, which are nanoscale *proteoliposomes* carrying PAMPs, proteins, and nucleic acids. Outer membrane vesicles from bacteria like *E. coli* have been shown to act as potent immune adjuvants, inducing the production of IFN-γ and the chemokine CXCL10 within the TME. This promotes robust infiltration of cytotoxic CD8^+^ T cells and synergizes with anti-PD-1 therapy. Conversely, specific virulence factors can co-opt host signaling to promote immune evasion.^164^^,^^165^ In gastric cancer, the *H. pylori* virulence factor CagA has been found to up-regulate PD-L1 expression in gastric cancer cell-derived exosomes, which in turn suppresses the activity of CD8^+^ T cells in the TME^148^ (Fig. 3D).

#### Microbial metabolites

The intra-tumoral microbiota can produce a vast and diverse array of metabolites. These small molecules act as critical signaling messengers that can reprogram the functions of both immune and cancer cells (Fig. 3E).

i) Short-chain fatty acids: Butyrate, a major short-chain fatty acid produced from the fermentation of dietary fiber by the gut microbiota, can promote the differentiation and function of FOXP3+ Tregs in the colon by inhibiting histone deacetylases (HDACs), thereby exerting an immunosuppressive effect.^166^ Conversely, under specific conditions, butyrate enhances the cytotoxic function of CD8^+^ T cells and up-regulates effector molecules like IFN-γ and granzyme B through the same HDAC-inhibitory mechanism, thus synergizing with oxaliplatin or anti-PD-L1 therapy. This reveals the complexity and precision of regulation by microbial metabolites.^167^^,^^168^
ii) Tryptophan metabolites and the aryl hydrocarbon receptor (AhR): Intra-tumoral *Lactobacillus reuteri* can metabolize dietary tryptophan to produce indole-3-aldehyde (I3A). As a ligand for the AhR, I3A can act directly on CD8^+^ T cells to promote their differentiation into an effector phenotype and enhance IFN-γ production, thereby effectively synergizing with ICI therapy to inhibit melanoma growth.^61^
iii) Other key metabolites: Inosine, produced by *Bifidobacterium pseudolongum*, has been found to activate the adenosine A2A receptor on T cells, promoting Th1 cell differentiation and enhancing the efficacy of checkpoint blockade.^169^ Trimethylamine N-oxide (TMAO), a metabolite associated with the order *Clostridiales* in triple-negative breast cancer, has been shown to enhance anti-tumor immunity by activating endoplasmic reticulum stress and inducing tumor cell pyroptosis, which in turn augments the CD8^+^ T cell response.^170^ In cervical cancer, L-lactate produced by tumor-resident *Lactobacillus* has been demonstrated to drive metabolic reprogramming that confers resistance to chemoradiotherapy by recruiting immunosuppressive immune cells.^55^

## Regulation of TME cell types by the intra-tumoral microbiota

The influence of the intra-tumoral microbiota on the TME extends far beyond triggering inflammation. It acts as a regulatory hub within a complex cellular network, coordinating multidimensional communication among cancer cells, immune cells, and stromal cells, thereby profoundly shaping tumor progression and therapeutic response.

### Cancer cell

#### Metabolic reprogramming and chemoresistance

Intra-tumoral microbiota play a critical role in the metabolic reprogramming of tumors, thereby promoting chemoresistance. Certain intra-tumoral bacteria can metabolize and inactivate chemotherapeutic drugs like gemcitabine by expressing specific enzymes, such as the cytidine deaminase secreted by *Gammaproteobacteria*, thereby conferring drug resistance.^105^ Similarly, *mycoplasmas* (*e.g.*, *Mycoplasma hyorhinis*) exacerbate chemoresistance by interfering with nucleotide metabolism and enhancing glycolysis.^171^ Antibiotic depletion experiments have confirmed a causal link between these bacteria and resistance; for instance, eliminating intra-tumoral bacteria can reverse gemcitabine resistance.^105^ Furthermore, *F. nucleatum* promotes chemoresistance by modulating autophagy to protect cancer cells from drug-induced damage.^172^ *H. pylori*, via its CagA protein, enhances the resistance of gastric cancer cells to 5-fluorouracil by up-regulating cellular glucose metabolism.^173^ Fungal microbiota trigger chemoresistance of cancer cells by enhancing interleukin-33 (IL-33) secretion.^107^ Viruses such as *HPV* can enhance resistance to chemotherapy through various mechanisms, including activating DNA repair and epithelial–mesenchymal transition pathways, promoting stem cell characteristics, and inhibiting apoptosis via their E6/E7 oncoproteins.^174^ The *HBV* X protein modulates autophagy and glucose metabolism, enhancing tolerance to chemotherapeutic drugs.^175^^,^^176^

#### Promoting proliferation, invasion, and metastasis

Intra-tumoral microbiota can directly stimulate cancer cell proliferation and migration by producing toxins (*e.g.*, colibactin from *E. coli* induces DNA double-strand breaks^177^) or expressing surface adhesins (*e.g.*, Fap2 from *F. nucleatum* activates Wnt/β-catenin signaling^178^). In breast cancer, bacteria such as *Staphylococcus*, *Lactobacillus*, and *Streptococcus* have been shown to modulate the actin cytoskeleton of cancer cells, thereby enhancing their metastatic potential.^78^ Oncoviral proteins (*e.g.*, *HPV* E6/E7, *EBV* LMP1, *HBV* HBx) drive malignant transformation and genomic instability by degrading tumor suppressors, activating survival signaling pathways, or causing insertional mutagenesis.^179^^,^^180^

### Immune cell

#### Myeloid cells (macrophages and MDSCs)

Bacteria within the TME can skew myeloid cell function towards a pro-tumorigenic state. In pancreatic cancer, an intra-tumoral bacterial community rich in Gram-negative species activates TLR2 and TLR4 on myeloid cells. This chronic stimulation polarizes macrophages towards an M2-like immunosuppressive phenotype and drives the expansion of MDSCs, ultimately promoting tumor progression.^104^ Similarly, *F. nucleatum* selectively recruits tumor-infiltrating myeloid cells, including tumor-associated macrophages and MDSCs, to the CRC microenvironment.^181^ These recruited cells suppress T cell activity by expressing arginase-1 and inducible nitric oxide synthase, thereby establishing a potent immunosuppressive niche.^182^ In esophageal squamous cell carcinoma, Gram-negative bacteria-derived LPS engages TLR4 receptors on tumor-associated macrophages, triggering downstream NF-κB signaling and promoting the transcription of immunosuppressive cytokines such as IL-10 and transforming growth factor-beta (TGF-β).^183^^,^^184^ This sustained activation turns macrophages toward an M2-like phenotype, characterized by high CD163 expression and potent immunosuppressive capacity, thereby dampening local anti-tumor immunity and correlating with poor prognosis.^183^ Similarly, spatial mapping studies of oral squamous cell carcinoma and CRC reveal that bacterial micro-niches are enriched for myeloid cells expressing arginase 1 (ARG1) and cytotoxic T lymphocyte antigen-4 (CTLA-4), while proliferative T cells are depleted.^29^ Single-cell transcriptomic analysis shows that bacteria-engulfing macrophages increase the production of chemokines such as CCL2, CCL4, and CXCL8, as well as inflammatory mediators via Janus kinase (JAK)–signal transducer and activator of transcription (STAT) and TNF pathways. This ultimately recruits more myeloid cells and strengthens an immunosuppressive microenvironment.^29^ Moreover, microbial metabolites such as short-chain fatty acids can further modulate macrophage epigenetics. For instance, butyrate inhibits HDACs in myeloid cells, suppressing pro-inflammatory gene expression and supporting M2 polarization.^167^ Collectively, these findings highlight the multifaceted regulatory role of intra-tumoral microbiota in shaping tumor-associated macrophages’ function and establishing a tumor-permissive niche.

#### Dendritic cell

Bacteria within the TME can profoundly shape DC function, thereby modulating anti-tumor immunity. Intra-tumoral bacterial taxa such as *Bifidobacterium* and *Akkermansia muciniphila* are capable of activating the cyclic GMP-AMP synthase-stimulator of IFN genes (cGAS–STING) pathway in DCs.^158^^,^^185^ This activation leads to a robust production of type I interferons, enhancing DC maturation and cross-presentation, and ultimately boosting CD8^+^ T cell responses. Moreover, bacterial PAMPs, including LPS and flagellin, can engage TLR2/4 on DCs, triggering the MyD88–NF-κB axis and promoting the secretion of pro-inflammatory cytokines such as IL-12.^155^^,^^186^ In addition to direct receptor stimulation, microbial metabolites are critical modulators of DC phenotype. Short-chain fatty acids, like butyrate, can alter DC cytokine profiles and antigen-presentation capacity via inhibition of HDACs, thereby influencing T cell polarization.^166^^,^^187^ Notably, bacterial-derived I3A produced by *Lactobacillus reuteri* can act on the AhR in DCs to shape T cell priming and enhance immune checkpoint blockade efficacy.^188^^,^^189^ Furthermore, certain intra-tumoral bacteria or their antigens may be processed and presented by DCs, directly stimulating tumor-infiltrating lymphocytes and contributing to molecular mimicry-based immune responses.^59^

#### T lymphocytes

Microbiota within the TME can orchestrate T lymphocyte dynamics, profoundly influencing tumor immunity and therapeutic outcomes. For instance, specific intra-tumoral microbiota such as *Lachnoclostridium*, *Blautia*, and *Faecalibacterium* can up-regulate chemokines like CXCL9, CXCL10, and CCL5, thereby promoting the recruitment and infiltration of CD8^+^ T cells into the tumor core and enhancing anti-tumor responses.^190^^,^^191^ Conversely, *F. nucleatum* has been shown to suppress T cell infiltration by increasing the expression of immunosuppressive molecules, including PD-1 and CTLA-4, and by promoting the accumulation of Tregs and MDSCs, leading to a “cold” tumor immune microenvironment and T cell exhaustion.^29^^,^^36^^,^^192^^,^^193^ At the molecular level, PAMPs such as LPS can activate TLR4 on antigen-presenting cells, triggering the MyD88–NF-κB pathway and modulating cytokine profiles that shape T cell polarization toward effector or regulatory phenotypes.^155^^,^^186^ Moreover, microbial metabolites such as short-chain fatty acids and indole derivatives further modulate T cell function. Butyrate, for example, can enhance CD8^+^ T cell memory and cytotoxicity via HDAC inhibition,^166^ while I3A activates the AhR pathway to boost tumor-infiltrating CD8^+^ T cell IFN-γ production and improve response to immune checkpoint blockade.^189^ Intriguingly, microbial antigens presented by tumor cells or DCs can also induce T cell cross-reactivity, as bacteria-derived peptides are recognized by tumor-infiltrating lymphocytes through molecular mimicry, potentially amplifying anti-tumor immune responses.^59^^,^^194^ Oncoviruses have evolved mechanisms to subvert T cell surveillance: high-risk *HPV* E5/E6/E7 proteins down-regulate MHC class I expression on cervical cancer cells to evade cytotoxic T cell recognition,^195^, ^196^, ^197^ while the *EBV* LMP1 protein up-regulates PD-L1 on nasopharyngeal carcinoma cells to directly inhibit T cell activity. Taken together, these findings highlight the complex and multifaceted regulatory influence of intra-tumoral microbiota on the recruitment, activation, and exhaustion of T cells within the TME.

### Stromal compartment

The intra-tumoral microbiota supports tumor progression by modulating stromal components to remodel the TME. Cancer-associated fibroblasts, as vital drivers, are responsible for ECM remodeling, growth factor secretion, and immunosuppression.^198^ Microbial products like bacterial LPS can activate the TLR4 pathway in fibroblasts, inducing their conversion to a pro-tumorigenic cancer-associated fibroblast phenotype that secretes factors like TGF-β, which promotes tumor growth and suppresses immune responses.^199^ Angiogenesis is also influenced by microbiota: attenuated *Salmonella typhimurium* exerts anti-tumor effects by inhibiting tumor vessel formation,^200^ whereas certain intra-tumoral microbial metabolites may promote neovascularization by up-regulating vascular endothelial growth factor (VEGF) expression.^201^^,^^202^ Furthermore, microbiota participate in disrupting the epithelial barrier; bacteria use proteases to degrade tight junction proteins, increasing tissue permeability, which not only facilitates microbial translocation but also promotes cancer cell invasion and metastasis.^203^

## Therapeutic applications of intra-tumoral microbiota in cancer treatment

Intra-tumoral microbiota, as a critical component of the tumor ecosystem, have gained increasing attention for their roles in tumor initiation, progression, and therapeutic response. Historical records from as early as the 13th century document cases where severe bacterial infections inhibited tumor progression.^204^ In the late 19th century, William Coley developed a vaccine using inactivated Streptococcus pyogenes and Serratia marcescens, successfully treating sarcoma patients and demonstrating the therapeutic potential of microorganisms in cancer treatment.^3^^,^^205^^,^^206^ Clinical studies indicate that antibiotic eradication of specific microorganisms may prevent tumorigenesis and enhance cancer therapies. For example, quadruple antibiotic regimens targeting *H. pylori* halt the progression from gastric epithelial dysplasia to gastric cancer.^207^^,^^208^ Antiviral therapies and vaccines represent critical intervention strategies, as exemplified by the prevention and treatment of HPV-associated cervical cancer,^209^ HBV-associated hepatocellular carcinoma,^210^ and *EBV*-associated nasopharyngeal carcinoma.^22^ Although research on intra-tumoral microbiota is still in its early stages, targeting and harnessing these microbial communities has emerged as a promising strategy for cancer prevention and treatment in current and future clinical paradigms.

### Engineered bacteria

Increasing evidence supports the therapeutic potential of directly injecting bacteria into tumor sites. Bacterial species, including *Clostridium*, *Salmonella*, *E. coli*, *Listeria*, and *Actinomyces*, have been widely studied and applied in cancer treatment due to their unique biological properties. For in-depth analysis, consider *Clostridium novyi-NT* as a significant example: this *anaerobic bacterium* is engineered to preferentially colonize hypoxic tumor regions, where it replicates and produces toxins that induce tumor necrosis while stimulating innate immune responses via PAMPs, leading to enhanced antigen presentation and T-cell infiltration.^211^ Early-phase clinical trials have demonstrated partial anti-tumor efficacy in solid tumors, such as refractory sarcomas, by remodeling the immunosuppressive TME.^212^

Among other strains, genetically engineered *Salmonella strains*, such as *VNP20009*, suppress tumor growth by impairing angiogenesis and remodeling the TME.^213^ Engineered *E. coli* has been used to deliver anti-tumor factors or therapeutic agents, enhancing localized immune responses.^214^ Similarly, genetically modified *Listeria strains*, such as ADXS11-001, activate antigen-presenting cells and stimulate tumor-specific T cell immunity. These approaches have shown promise in *HPV*-associated malignancies, including cervical cancer.^215^^,^^216^ Additionally, Actinomyces species, including the *Bacillus Calmette-Guérin* vaccine, have been widely used in bladder cancer treatment, reducing tumor recurrence rates through localized immune activation.^217^^,^^218^ Other bacterial species have been engineered to express anti-tumor factors or metabolites, leveraging oncolytic and immune-modulatory mechanisms to expand microbial-based anti-cancer strategies.^219^

Despite the promising preclinical results, the translation of engineered bacteria into clinical practice is fraught with significant challenges. Toxicity is a primary concern, particularly with Gram-negative bacteria such as *Salmonella* and *E. coli*, since their LPS component can trigger a potent inflammatory response, which could result in septic shock.^213^^,^^219^ Therefore, extensive attenuation is required to balance safety and efficacy. Another major obstacle is the regulatory hurdles involved. Engineered bacteria are classified as live biotherapeutic products, a category with which regulatory bodies such as the FDA are relatively unfamiliar.^220^^,^^221^ This creates uncertainty regarding manufacturing requirements (*e.g.*, ensuring the genetic stability of engineered plasmids), quality control, and clinical trial design.^220^^,^^221^ Finally, ensuring bacterial containment within the tumor and preventing off-target dissemination or colonization remains a critical safety and engineering challenge that must be addressed for widespread clinical adoption.^219^^,^^222^

### Antibiotics

Recent studies indicate that targeting intra-tumoral microbiota with antibiotics represents a potential anti-cancer strategy. In CRC, *F. nucleatum* drives tumor progression by mediating inflammatory activation, immune suppression, and chemoresistance. Antibiotics such as metronidazole significantly reduce *F. nucleatum* abundance, inhibiting tumor growth and restoring chemosensitivity.^7^^,^^172^ In gastric cancer, *H. pylori* eradication reduces gastric cancer incidence and gastric mucosa-associated lymphoid tissue (MALT) lymphoma lesion size.^223^^,^^224^ Pancreatic cancer studies show that intra-tumoral microbial communities, including *Gammaproteobacteria*, metabolize gemcitabine, conferring drug resistance. Broad-spectrum antibiotics enhance chemotherapy efficacy and improve immunotherapy responses.^104^^,^^105^ In breast and liver cancers, antibiotics targeting specific bacteria, including *Staphylococcus* and *Clostridium* species, mitigate pro-inflammatory microenvironments and suppress tumor progression.^136^^,^^225^ Notably, emerging evidence from a murine spontaneous breast-tumor model (MMTV-PyMT) and human breast cancer samples highlights the role of tumor-resident intracellular microbiota, primarily Firmicutes genera such as *Staphylococcus*, *Lactobacillus*, *Enterococcus*, and *Streptococcus*, in promoting metastatic colonization without affecting primary tumor growth. Depletion of intra-tumor bacteria via targeted antibiotics significantly reduces lung metastasis in breast cancer models by impairing CTC viability, while sparing gut microbiota and primary tumor growth.^78^

However, antibiotic use raises concerns about dysbiosis and antimicrobial resistance development. Clinical studies demonstrate that systemic antibiotic administration significantly reduces ICI efficacy, primarily by impairing gut microbiota diversity and functionality, thus compromising host anti-tumor immune responses. A major research challenge is selectively targeting intra-tumoral bacteria while preserving the gut microbiome. One promising strategy employs nanomaterials as drug delivery vehicles for precise antibiotic transport to tumor tissues, showing preliminary success in mouse models of colorectal and breast cancers.^226^^,^^227^ Future research should prioritize understanding the dynamic interplay between antibiotics and the microbiota–immune axis, providing novel theoretical foundations and therapeutic strategies for personalized tumor immunotherapy.

### Bacteriophage

Bacteriophages are viruses that specifically infect bacteria, exploiting host resources for replication and ultimately lysing bacterial cells to release progeny phages. Bacteriophage-based tumor therapy has emerged as a novel strategy with significant therapeutic potential. For in-depth analysis, consider phages targeting *F. nucleatum* as a main example: these phages selectively lyse *F. nucleatum* in CRC, disrupting the immunosuppressive TME by reducing MDSC recruitment and enhancing chemotherapy efficacy through restored immune activation.^228^ Bacteriophages can also serve as efficient drug delivery vehicles, precisely delivering chemotherapeutic agents, gene-editing tools, or immunomodulators through surface modifications, improving therapeutic outcomes while minimizing systemic toxicity.^229^, ^230^, ^231^, ^232^, ^233^, ^234^

Bacteriophages exhibit high immunogenicity, with capsid proteins directly stimulating the host immune system, inducing DC maturation, and promoting tumor-associated macrophage polarization toward the M1 phenotype. This process up-regulates M1-type cytokines, including TNF-α and IL-6, recruiting neutrophils, and enhancing tumor cytotoxicity. Combined with ICIs, bacteriophage therapy may amplify tumor-specific immune responses. Bacteriophages can inhibit angiogenesis by targeting tumor endothelial cells or carry anti-cancer genes to induce tumor cell apoptosis, expanding their therapeutic applications.^233^^,^^235^^,^^236^

Although bacteriophage-based tumor therapy remains in the experimental stage, it offers several advantages, including high specificity, low toxicity, and multifunctionality.^237^ However, significant challenges must be overcome for this therapy to progress from laboratory research to clinical practice. One major obstacle is immunogenicity: the host immune system can recognize phages as foreign and produce neutralizing antibodies that clear them from circulation rapidly.^238^ This limits therapeutic efficacy, particularly with repeated administration.^238^ Barriers to delivery also pose a problem, as systemic administration struggles to achieve sufficient phage concentration in tumors due to clearance by the immune system and physical barriers such as the tumor stroma.^239^^,^^240^ Finally, the regulatory landscape for phage therapy is complex and not yet fully established. Issues such as ensuring the purity of phage preparations (*i.e.*, the absence of bacterial toxins), managing the potential horizontal transfer of undesirable genes, and defining appropriate manufacturing and quality control standards present significant regulatory challenges, requiring close collaboration between researchers and regulatory agencies.^241^

### Oncolytic virus

Oncolytic viruses (OVs) are a class of viruses that exhibit an inherent or engineered ability to target cancer cells, allowing them to destroy malignant cells while leaving normal tissues unharmed. OVs have multiple anti-tumor mechanisms. Firstly, OVs can directly induce tumor cell lysis through viral replication.^242^^,^^243^ Secondly, viral infection and subsequent cell death lead to the release of tumor-associated antigens and damage-associated molecular patterns (DAMPs), thereby eliciting robust innate and adaptive anti-tumor immune responses.^243^^,^^244^ This immunogenic cell death can turn “cold” tumors into “hot” tumors, which are characterized by increased immune cell infiltration and activity.^245^ Furthermore, certain OVs can disrupt tumor angiogenesis, thereby inhibiting tumor progression.^246^

OVs can be broadly grouped into several types based on their replication capacity. Some OVs, such as wild-type *reovirus*, have an intrinsic ability to replicate and exploit molecular abnormalities commonly found in cancer cells.^242^ More frequently, however, OVs are engineered through genetic modification. These strategies can generate replication-selective viruses, as demonstrated by talimogene laherparepvec (T-VEC): a genetically modified herpes simplex virus type 1 (*HSV-1*) which replicates preferentially within tumor cells.^247^^,^^248^ Alternatively, replication-defective vectors can be constructed. These vectors cannot produce new infectious progeny, but they can still deliver therapeutic payloads or induce immunogenic cell death.^242^

*Vesicular stomatitis virus* (VSV) is a prototypical example of an engineered viral vector.^249^ VSV is an RNA virus that is highly sensitive to type I IFN-mediated antiviral responses. Many cancer cells have defects in their IFN signaling pathways, which impair their ability to mount effective antiviral defense mechanisms and render them susceptible to VSV infection.^249^^,^^250^ In contrast, healthy cells with intact IFN responses can efficiently clear the virus, providing a natural basis for tumor selectivity. Furthermore, VSV can be genetically engineered to enhance its safety and therapeutic efficacy. For example, the VSV-ΔG variant has a deletion in the glycoprotein (G) gene, resulting in a virus that cannot replicate and has markedly reduced neurotoxicity.^251^ This platform enables the construction of pseudotyped viruses, whereby the G protein is replaced with glycoproteins from other viruses to alter viral tropism and circumvent pre-existing immunity.^251^^,^^252^

Several OVs have gained regulatory approval. T-VEC (Imlygic®), which expresses GM-CSF to boost systemic immunity, was approved by the US FDA in 2015 for advanced melanoma.^247^ H101 (Oncorine®), an engineered adenovirus, has been approved in China for treating head and neck cancer.^253^ G47Δ (Delytact®), a third-generation oncolytic *HSV-1*, has been approved in Japan for treating glioblastoma.^254^ Clinical trials combining OVs with ICIs have demonstrated encouraging synergistic effects, as OVs can remodel the TME and increase PD-L1 expression, making tumors more susceptible to anti-PD-1/PD-L1 therapy.^245^^,^^255^

The application of OVs is still facing numerous technical challenges and obstacles. The primary method of administering OVs is intra-tumoral injection, which restricts their use to patients with accessible tumors. Although intravenous delivery offers broader applicability, it is severely restricted by poor biodistribution.^256^^,^^257^ OVs are rapidly cleared by the reticuloendothelial system and inactivated by pre-existing or therapy-induced antiviral antibodies, which prevent sufficient amounts of the virus from reaching the tumor site effectively.^242^^,^^256^ In addition, excessive inflammation and off-target toxicity remain significant concerns. For instance, in brain tumors, the robust inflammatory responses triggered by OVs can result in severe, life-threatening neurotoxicity and cerebral edema, which poses a critical barrier to glioblastoma treatment.^254^ Overcoming these challenges through advanced delivery strategies, such as cell-based carriers or polymer coatings, as well as precise viral engineering to modulate immunogenicity, is essential for the future success of oncolytic virotherapy.^256^^,^^258^

## Current challenges and future perspectives

### Current challenges

The burgeoning field of intratumoral microbiota has fundamentally transformed our understanding of the complexity that shapes the TME. It has shifted the paradigm from viewing tumors as isolated lesions to recognizing them as intricate ecological systems. However, using intratumoral microbiota for cancer therapy and translating these advances into clinical practice still face several significant challenges.

### Technical challenges

A major technical challenge in intratumoral microbiome research is the low biomass of microbial communities, which results in a suboptimal signal-to-noise ratio. The scarcity of microbial DNA in these samples makes them highly susceptible to contamination from laboratory reagents, environmental sources, and host genomic material. This can result in false-positive findings. However, significant progress has been made in recent years with the development of bioinformatics tools such as Kraken2 and PathSeq,^118^ which enable contamination correction.

Future breakthroughs should focus on two areas: (1) establishing a comprehensive, standardized workflow for sample collection and sequencing that incorporates the use of ultra-clean reagents and systematic negative controls, and (2) integrating high-resolution fluorescence *in situ* hybridization (FISH) with spatial multi-omics technologies to enable the *in situ* visualization and spatial mapping of microbiota within the TME. These advances will enable the direct validation of three-dimensional interaction networks among microbiota, tumor cells, and immune cells, providing robust evidence for mechanistic studies.

#### From correlation to causation

Currently, most studies are cross-sectional in nature, primarily revealing associations between microbial features and tumors. However, these studies are insufficient to address the pivotal question of causality: do specific microbes cause tumor formation, or does the altered TME select for particular microbial colonization? This uncertainty remains a major barrier to developing targeted therapies.

Elucidating the causal relationship between intra-tumoral microbiota and tumor immunity requires multidisciplinary research approaches. Large-scale, longitudinal cohort studies can monitor the dynamic interplay between the microbiome and tumor evolution. Meanwhile, causal inference models, such as Mendelian randomization, can use host genetic variation to evaluate the potential impact of the microbiome on cancer risk. In terms of mechanism, colonizing germ-free animal models with defined microbial communities, alongside patient-derived xenograft and organoid technologies, can provide direct experimental evidence of the role of microbiota in tumor initiation, progression, and therapeutic response.

### Clinical translation

Despite the significant translational potential of intra-tumoral microbiota, there are multiple challenges to its clinical application. The high degree of heterogeneity in microbial communities, which is influenced by genetic background, dietary habits, and geographical factors, limits the development of universal biomarkers and therapeutic targets. Furthermore, progress in live biotherapeutics, engineered bacteria, and phage therapies must overcome evolving regulatory landscapes and technological constraints.

### Future perspectives

The intra-tumoral microbiome has emerged as a novel frontier in tumor biology. Although the path from basic research to clinical application remains challenging, this field has significant potential to advance the next generation of precision oncology. Future research will likely focus on several key directions.

#### Microbiome-based multi-modal precision diagnosis and therapy

The composition of intra-tumoral microbiota shows significant heterogeneity between patients. A core task moving forward is to integrate multi-omics datasets, including microbial metagenomics/transcriptomics, host genomics, immune microenvironment profiling, and clinicopathological information, to construct high-precision, multimodal predictive models. These models will facilitate the prediction of patient responses to existing therapies, such as ICIs, and guide personalized microbial intervention strategies. For instance, in tumors such as CRC, which are enriched with certain pro-tumorigenic bacteria (*e.g.*, *F. nucleatum*), highly specific antimicrobials or phage therapies could be developed to target and eradicate these bacteria. Conversely, genetically engineered probiotics could be used as “living medicines” to deliver cytokines, immune agonists, or tumor antigens *in situ* to reprogram the immunosuppressive microenvironment and convert “cold” tumors into “hot” tumors, thereby enhancing anti-tumor immunity.

#### Delivery systems

The precise delivery of microbial agents or related interventions to tumor sites is essential to realize their therapeutic potential while minimizing systemic toxicity. Therefore, the development of smart drug delivery systems that can efficiently target the TME is essential. One future approach is to encapsulate antibiotics or phages in nanocarriers, such as liposomes or polymeric nanoparticles, to exploit the enhanced permeability and retention effect and achieve passive and active targeting via conjugation with tumor-specific ligands. Natural carriers, such as exosomes or engineered bacterial outer membrane vesicles, are also being explored as delivery vehicles to improve biocompatibility and reduce immunogenicity. These advanced platforms aim to increase drug concentrations within tumors while reducing off-target effects on healthy tissues and commensal microbiota, thereby increasing the therapeutic window for microbial therapies.

#### Safety and regulation of microbial therapies

The safety risks associated with live microbial therapies, particularly those involving Gram-negative strains, cannot be overlooked. Bacterial components such as LPS can trigger uncontrolled inflammatory responses and even sepsis. Therefore, future research must establish rigorous safety evaluation systems, including: i) the selection and identification of strains with well-documented safety profiles; ii) genetic engineering approaches to reduce virulence, such as creating auxotrophic strains that depend on tumor-specific metabolites for survival, or integrating safety control circuits, such as “kill switches”.

Another pressing challenge is the lack of standardized research protocols and reporting guidelines, which impedes reproducibility and obscures the path to clinical translation. The urgent need for internationally recognized standard operating procedures encompasses sample collection and processing, sequencing workflows, data interpretation, and clinical trial design. Close collaboration between regulatory agencies, researchers, and industry stakeholders is required to address key issues such as the design of clinical trials, data privacy, ethical standards, and long-term safety supervision, thereby paving the way for the healthy development and eventual clinical implementation of microbial therapies.

## Conclusion

Despite considerable challenges, research into the intra-tumoral microbiome is pioneering new frontiers in cancer biology and therapeutics. The synergistic integration of technological innovation, mechanistic studies, and clinical translation means that intra-tumoral microbiota are set to be a breakthrough in precision oncology, beginning a new era of personalized cancer treatment.

## CRediT authorship contribution statement

**Junju He:** Writing – original draft, Software, Methodology, Investigation, Formal analysis, Data curation, Conceptualization. **Hui Tan:** Writing – original draft, Data curation, Conceptualization. **Yanru Qiu:** Writing – review & editing, Data curation. **Yuchao Dan:** Validation, Resources, Data curation. **Qian Wan:** Resources, Investigation, Data curation. **Lan Li:** Data curation, Conceptualization. **Jie Wu:** Formal analysis, Data curation. **Qibin Song:** Writing – review & editing, Supervision, Conceptualization. **Hongbin Chen:** Writing – review & editing, Supervision, Conceptualization. **Bin Xu:** Writing – review & editing, Supervision, Project administration, Funding acquisition, Conceptualization.

## Data availability

Any additional information required to reanalyze the data reported in this paper is available from the lead contact upon request.

## Declaration of generative AI and AI-assisted technologies in the writing process

During the preparation of the original draft of this work, the authors used the free version of OpenAI-ChatGPT c2022 (v.GPT-4o, Jan 2024) to improve manuscript readability and language. After using this tool, the authors thoroughly reviewed and modified the manuscript and take full responsibility for the content of the publication. The literature survey was done only by the authors, with no input from the AI tools.

## Funding

This work was funded by the 10.13039/501100001809National Natural Science Foundation of China (No. 82403850, 82203502), the Cross-Innovation Talent Project of 10.13039/501100016345Renmin Hospital of Wuhan University (China) (No. JCRCGW-2022-002), and the 10.13039/501100003819Natural Science Foundation of Hubei Province, China (No. 2021CFB086).

## Conflict of interests

The authors declared no conflict of interests.
